# Effect of thyroid statuses on sodium/iodide symporter (*NIS*) gene expression in the extrathyroidal tissues in mice

**DOI:** 10.1186/1756-6614-3-3

**Published:** 2010-06-09

**Authors:** Harun-Or-Rashid, Masato Asai, Xiao-yang Sun, Yoshitaka Hayashi, Junichi Sakamoto, Yoshiharu Murata

**Affiliations:** 1 Research Institute of Environmental Medicine, Nagoya University Graduate School of Medicine, Nagoya, Japan; 2 Department of Healthcare Administration, Nagoya University Graduate School of Medicine, Nagoya, Japan; 3 Department of Pathology, Nagoya University Graduate School of Medicine, Nagoya, Japan

## Abstract

**Background:**

Iodide that is essential for thyroid hormone synthesis is actively transported into the thyroid follicular cells via sodium/iodide symporter (NIS) protein in vertebrates. It is well known that NIS expression in thyroid is regulated by the thyroid statuses mainly through thyroid stimulating hormone (TSH). Although *NIS *mRNA expressions in extrathyroidal tissues have been qualitatively reported, their regulation by thyroid statuses has not been well clarified.

**Methods:**

Male ICR mice aged four weeks were assigned into three groups (control, hypothyroid, and hyperthyroid). Hypothyroid group of mice were treated with 0.02% methimazole in drinking water and hyperthyroid group of mice received intraperitoneal injection (4 μg _L_-T_4 _twice a week) for four weeks. *NIS *mRNA expression levels in the tissues were evaluated using Northern blot hybridization and quantitative real-time RTPCR (qPCR). Additionally, end-point RTPCR for the thyroid follicular cell-characteristic genes (TSH receptor, *TSHR*; thyroid transcription factor-1, *TTF1*; and paired box gene 8, *Pax8*) was carried out.

**Results:**

By Northern blot analysis, *NIS *mRNA was detected in thyroid and stomach. In addition to these organs, qPCR revealed the expression also in the submandibular gland, colon, testis, and lung. Expression of *NIS *mRNA in thyroid was significantly increased in hypothyroid and decreased in hyperthyroid group. Trends of *NIS *mRNA expression in extrathyroidal tissues were not in line with that in the thyroid gland in different thyroid statuses. Only in lung, *NIS *mRNA was regulated by thyroid statuses but in opposite way compared to the manner in the thyroid gland. There were no extrathyroidal tissues that expressed all three characteristic genes of thyroid follicular cells.

**Conclusions:**

*NIS *mRNA expression in the thyroid gland was up-regulated in hypothyroid mice and was down-regulated in hyperthyroid mice, suggesting that *NIS *mRNA in the thyroid gland is regulated by thyroid statuses. In contrast, *NIS *mRNA expression in extrathyroidal tissues was not altered by thyroid statuses although it was widely expressed. Lack of responsiveness of *NIS *mRNA expressions in extrathyroidal tissues reemphasizes additional functions of NIS protein in extrathyroidal tissues other than iodide trapping.

## Background

Thyroid hormone plays a central role in determining the levels of thermogenesis and basal metabolism. The sodium-iodide symporter (Na^+^/I^- ^symporter, NIS) plays pivotal role in iodide condensation that is an indispensable step in the biosynthesis of the thyroid hormone. *NIS *cDNA has been cloned in various species including rat [1], human [2] and mouse [3]. Physiological importance of NIS was confirmed by the identification of *NIS *mutations in patients who had congenital hypothyroidism due to lack of iodide transport [4,5].

Regulation of *NIS *mRNA in the thyroid follicular cells has been investigated both *in vivo *and *in vitro*. Levy *et al. *showed that NIS protein was drastically increased in hypothyroid rats [6]. They also demonstrated that that up-regulation of NIS expression was diminished by hypophysectomy and that thyroid stimulating hormone (TSH) administration restored NIS expression. Saito *et al. *also showed that NIS activity was increased by TSH or forskolin in primary cultured human thyroid cells [7]. These results clearly demonstrated that cAMP/PKA pathway provoked by TSH/TSH receptor (TSHR) plays an essential role in control of NIS expression in thyroid.

Several transcription factors have been shown to play an important role in thyroid-specific expression of NIS. Endo *et al. *demonstrated that thyroid transcription factor-1 (TTF1), alternatively known as Nkx2-1 (homeobox protein Nkx2-1), activates the promoter activity of rat *NIS *gene [8]. Ohno *et al. *showed that paired box gene 8 (Pax8) binds and activates upstream enhancer in rat *NIS *gene [9].

Interestingly, *NIS *gene is expressed in a wide range of extrathyroidal tissues, including mammary gland, colon, ovary, [10], salivary glands, and gastric mucosa [11]. Among those NIS-positive tissues, the active iodide transport via NIS protein has been confirmed in salivary glands, gastric mucosa, lactating mammary gland, and intestine [12-14].

Not only the normal tissues, but also certain types of cancer cells are expressing NIS. For example, *NIS *mRNA is detected in most thyroid cancer specimens [15]. Besides thyroid cancer cells, NIS is predominantly expressed intracellularly in many other carcinomas including breast cancer, lung cancer, salivary gland cancer, colon cancer, prostate cancer, and stomach cancer etc. [16].

As mentioned above, NIS expression and its regulation were well studied in normal thyroid cells. However, quantitative analysis of *NIS *mRNA and transcriptional regulation of *NIS *gene by thyroid statuses in extrathyroidal tissues have not been explored. Therefore, in the present study, we analyzed expression of *NIS *mRNA in various tissues in different thyroid statuses.

## Methods

### Animal experiments

Male ICR mice aged four weeks were obtained from Japan SLC, Inc. (Japan) and randomly divided into three groups. Three or four mice were housed in each cage under controlled conditions of temperature and light. Mice in hyperthyroid group (n = 7) were intraperitoneally injected with solution containing 4 μg L-thyroxine (_L_-T_4_) twice a week. Mice in control group (n = 7) and hypothyroid group (n = 7) were injected with vehicle twice a week. Mice in hypothyroid group were given 0.02% methimazole as drinking water and the other mice were given regular tap water. Treatment was continued for four weeks until all the mice were sacrificed. Tissues (the thyroid gland, submandibular gland, pituitary, lung, liver, stomach, colon, testis, and pancreas) were immediately frozen after the dissection and stored at -80°C. Dissection of small organs (the thyroid gland, pituitary) was performed under the dissecting microscope (Stereomicroscope SZX7, Olympus, Tokyo, Japan). All the experiments were carried out in accordance with the principles and procedures outlined by the Committee for Animal Experiment of Nagoya University School of Medicine and Research Institute of Environmental Medicine.

### RNA isolation and cDNA synthesis

Total RNA was isolated from approximately 50 mg frozen tissue by using TRIzol (Invitrogen, Carlsbad, CA) according to the manufacturer's protocol. Extracted RNA samples were treated with DNase (Qiagen, Valencia, CA), followed by purification using RNeasy Mini kit (Qiagen). 500 ng RNA of each sample was reverse-transcribed in 20 μl reaction volume using ReverTra Ace (Toyobo Co., LTD., Japan), and the cDNA samples were diluted 20-times with nuclease-free water for both end-point RTPCR and quantitative real-time RTPCR (qPCR).

### Gene expression analyses

Northern blot analysis was done according to the protocol described previously in detail [17]. For both end-point RTPCR and qPCR, 25 ng purified RNA-equivalent cDNA was used as a template. End-point RTPCR was performed with GeneAmp PCR system 9700 (Applied Biosystems, Warrington, UK) and Blend Taq (Toyobo) using a program: 94°C for 2 min, [94°C for 10 sec, 60°C for 40 sec] × 35 cycles, 72°C for 7 min. PCR products were separated on 2% agarose gel and visualized with ethidium bromide. qPCR was performed with the ABI-PRISM 7000 Sequence Detector System (Applied Biosystems) and Power SYBR Green (Applied Biosystems) using a program: 95°C for 3 min, [95°C for 10 sec, 60°C for 40 sec] × 40 cycles. Expression of each target gene was measured in duplicate and was normalized relative to 18S ribosomal RNA expression. Primers for the qPCR of mouse *NIS *gene were 5'-AGCAGGCTTAGCTGTATCCC-3' (forward) and 5'-AGCCCCGTAGTAGAGATAGGAG-3' (reverse) to yield 235 bp products. Mouse *18S *ribosomal RNA was measured as an internal control using following primers: 5'-GTAACCCGTTGAACCCCATTCGTGATG-3' (forward) and 5'-CGATCCGAGGGCCTCACTA-3' (reverse) to yield 172 bp. Likewise, the end-point RTPCR were done using following primers: for TSH receptor (*TSHR*), 5'-CTGCGGGGCAAAGAGTGTGC-3' (forward) and 5'-AGGGGAGCTCTGTCAAGGCA-3' (reverse) to yield 325 bp; for thyroid transcription factor-1 (*TTF1*), 5'-ATCTGAGCTGGGGTGCTGGG-3' (forward) and 5'-GCCCTGTCTGTACGCTGCGA-3' (reverse) to yield 244 bp; for paired box gene 8 (*Pax8*), 5'-CGGCGATGCCTCACAACTCG-3' (forward) and 5'-CCGGATGCTGCCAGTCTCGT-3' (reverse) to yield 221 bp. Gene accession numbers for the genes dealt in this paper are as follows: mouse *NIS *(sodium/iodide symporter), also known as *Slc5a5 *(solute carrier family 5, member 5) gene, NM_053248; mouse *18S *(18S ribosomal RNA), NR_003278; mouse *TSHR *(thyroid stimulating hormone receptor), NM_011648; mouse *TTF1 *(thyroid transcription factor-1), also known as *Nkx2-1 *(homeobox protein Nkx2-1), NM_009385 and *Pax8 *(paired box gene 8), NM_011040.

### Statistical analysis

Values of qPCR were expressed as mean ± SEM and were analyzed using unpaired t-test. A p value of < 0.05 was considered significant. All the qPCR experiments were repeated for at least three times, using at least three animals per group. Statistical analysis was done using Statview 5.0 (SAS institute Inc., Cary, NC).

## Results

### *NIS *mRNA expression in the thyroid gland and extrathyroidal tissues

**Figure 1:**
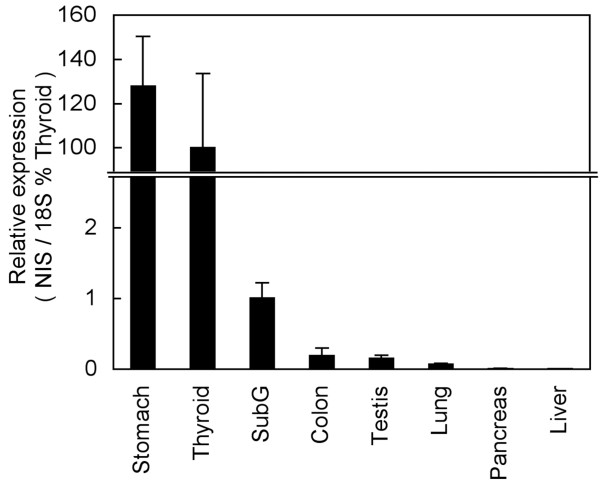
**Comparative tissue distributions of *NIS *mRNA**. *NIS *mRNA levels were estimated by quantitative real-time RTPCR (qPCR) experiments and standardized in respect to *18S *mRNA expression. Data are presented as a percentage of the value obtained for thyroid gland. 'SubG' in the panel stands for the submandibular gland.

To determine the distribution of *NIS *mRNA expression in control mice, qPCR was performed using RNA extracted from collected tissues and gene specific primers. Sample without cDNA was used as a negative control. The relative levels of *NIS *mRNA expression were as follows in descending order: stomach, thyroid, submandibular gland, colon, testis, and lung. Abundance of standardized *NIS *expression in stomach was as large as that in the thyroid gland (Figure 1).

**Figure 2:**
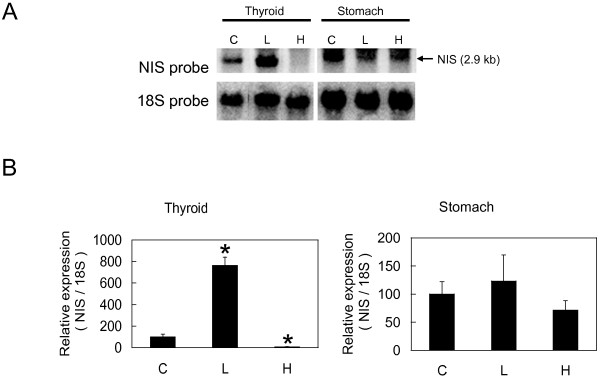
***NIS *mRNA regulation in different thyroid statuses in *NIS *abundant tissues**. A. Expression of *NIS *mRNA through Northern blot analysis. Total RNA (8 μg for thyroid and 15 μg for stomach) from animals under different thyroid statuses was loaded on 0.8% agarose gel, transferred to the Nylon membrane and hybridized to a ^32^P-labeled *NIS *specific DNA probe (upper lane). The same membrane used in the upper lane was rehybridized to *18S *probe to validate the consistency of loaded amount of RNA. B. Expression of *NIS *mRNA through quantitative real-time RTPCR (qPCR). 25 ng purified RNA-equivalent cDNA was amplified in one reaction. Expression of each target gene (thyroid, left panel; stomach, right panel) was measured in duplicate and was normalized relative to *18S *ribosomal RNA expression. Results are shown as mean ± SEM. * P < 0.05 compared with control (unpaired *t*-test). Control, C; hypothyroid, L; and hyperthyroid, H.

**Figure 3:**
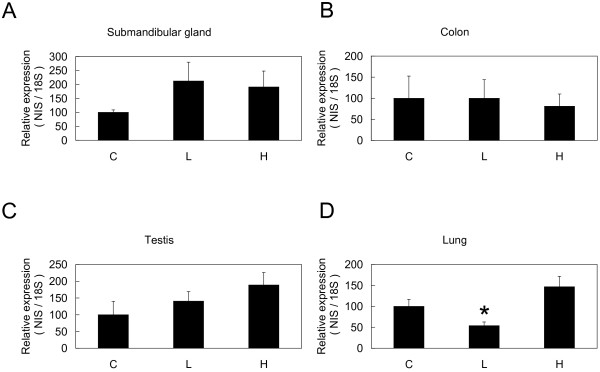
***NIS *mRNA regulation in different thyroid statuses in *NIS *scarce tissues**. Quantitative comparison of *NIS *mRNA in different thyroid statuses was performed in the same way as in Figure 2B using extrathyroidal tissues including submandibular gland (A), colon (B), testis (C), and lung (D). Results are shown as mean ± SEM. * P < 0.05 compared with control (unpaired *t*-test). Control, C; hypothyroid, L; and hyperthyroid, H.

To analyze the effects of thyroid statuses on NIS expression, we compared *NIS *mRNA expression in mice under three different thyroid statuses using Northern blot and qPCR. Northern blot analysis showed drastic changes of *NIS *mRNA expression in thyroid but not in stomach (Figure 2A). We also analyzed *TSHβ *mRNA expression in pituitary by qPCR and confirmed that *TSHβ *mRNA was up-regulated in hypothyroid group and down-regulated in hyperthyroid group (data not shown). To further quantify *NIS *mRNA expression in thyroid, qPCR was performed and statistically significant differences among control, hyperthyroid, and hypothyroid groups were confirmed in thyroid but not in stomach (Figure 2B). We then analyzed expression of *NIS *mRNA in extrathyroidal tissues using qPCR. Among *NIS *mRNA positive extrathyroidal tissues, no significant up- or down-regulation was observed in submandibular gland, colon, and testis (Figure 3A, B, and 3C). Although the expression levels were very low, *NIS *mRNA expression in lung was significantly suppressed in hypothyroid group compared with control group (Figure 3D).

### Expression of *TSHR, TTF1 *and *Pax8 *mRNA in the extrathyroidal tissues

**Figure 4:**
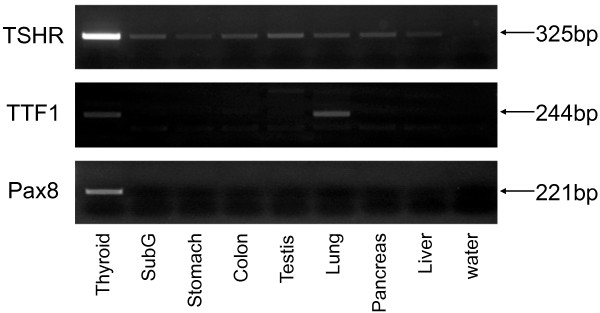
**Profiling of mRNA expression of thyroid follicular cell-characteristic genes**. End-point RTPCR was performed using cDNA from the thyroid gland and the extrathyroidal tissues using specific primers for thyroid stimulating hormone receptor (*TSHR*), thyroid transcription factor-1 (*TTF1*) and paired box gene 8 (*Pax8*). 'SubG' in the panel stands for the submandibular gland.

To probe the reason why no extrathyroidal tissues responded to thyroid statuses like thyroid, we added our study with profiling of genes that are considered characteristic to the thyroid follicular cells. We carried out end-point RTPCR for thyroid stimulating hormone receptor (*TSHR*), thyroid transcription factor-1 (*TTF1*, also known as homeobox protein *NKx2-1*), and paired box gene 8 (*Pax8*) using gene specific primers. While *TSHR *showed relatively broad mRNA expression in tested extrathyroidal tissues, expression of *TTF1 *mRNA was observed only in the lung, and no tissue was observed to express *Pax8 *mRNA (Figure 4).

## Discussion

In the present study, we analyzed expression levels of *NIS *mRNA in differential thyroid statuses in the mice extrathyroidal tissues for the first time. *NIS *mRNA expression in various mouse tissues has been analyzed by Perron *et al*. using densitometry of band images of end-point RTPCR product [3]. Our results are, in general, in agreements with their findings except for lung, for which very low but significant expression levels could be detected by qPCR in the present study.

We analyzed effect of thyroid statuses on *NIS *mRNA expression in mouse extrathyroidal tissues, and demonstrated that *NIS *mRNA in extrathyroidal tissues is regulated in a differential manner from that in thyroid. Our findings agree well with Brown's old remark in 1961 that unlike the thyroid gland, however, these extrathyroidal iodide-concentrating mechanisms have not been shown to respond to such procedures as hypophysectomy and thyroxine or TSH administration not only in rodents but in many vertebrates [18]. More recent paper pointed that iodide accumulation is not regulated by TSH in extrathyroidal tissues in rat [12].

Therefore, NIS protein in extrathyroidal tissues is unlikely to play important role in the thyroid hormone homeostasis, and physiological role of extrathyroidal NIS remains to be elucidated. Yet, extrathyroidal tissues: salivary glands, gastric mucosa, lactating mammary gland, choroid plexus, and the ciliary body of the eye; are reported to actively accumulate iodide through NIS [19]. While no extrathyroidal tissues have the ability to organify the iodide, role of NIS protein in extrathyroidal tissues may be to pool or to transfer the iodide. Another possible role of NIS protein is to transport molecules other than iodide. Due to the similarity in size and charge to iodide, thiocyanate or other anions are translocated in extrathyroidal tissues [19].

Only in lung, in which most feeble signals of *NIS *mRNA were detected, *NIS *mRNA was regulated in the opposite manner as that in the thyroid gland. Intriguingly, iodide has been used to thin mucus secretions in the respiratory tract as an expectorant drug for asthma or other pulmonary diseases [20]. It was also found that orally administered radio-iodide reaches bronchial lumen [21]. This transport of iodide across the bronchial mucosa may be conducted by NIS. Although we found thyroid follicular cell-characteristic gene, *TTF1 *mRNA expression in lung, there is no evidence to prove the cellular co-localization of NIS and TTF1 until a sensitive histological assay will be developed. As our *NIS *mRNA detection system using qPCR is extremely sensitive, this result may not mean the existence of functioning NIS protein in lung. Further investigation especially regarding the distribution of NIS positive cells in lung would be required to find physiological functions of NIS in lung. In addition, proper animal model (*i.e. *NIS null mice) would be helpful for the further understanding of functional role of NIS protein in the extrathyroidal tissues.

To offer a rationale for general lack of responsiveness to thyroid statuses in extrathyroidal *NIS *mRNA, we performed end-point RTPCR for *TSHR*, *TTF1*, and *Pax8 *mRNA. Table 1 shows, *TSHR *mRNA levels were the highest in thyroid. However, at low level, *TSHR *mRNA seems ubiquitously among tested tissues. On the other hand, *TTF1 *and *Pax8 *mRNA seem more thyroid-specific. Especially, *Pax8 *was expressed only in thyroid in this study. Lacks of TTF1 and/or Pax8 mRNA expression are likely the cause of insensitivity to thyroid status. We found that thyroid was the only tissue to express all the three genes, which may explain the strong *NIS *mRNA expression and tight regulation by thyroid statuses. To clarify the mechanism of *NIS *mRNA regulation and to know the physiological reason of its existence in extrathyroidal tissues, further investigation would be required.

**Table 1:** Expression of *NIS *in different tissues and its regulation by different thyroid statuses

	*NIS*	*TSHR*	*TTF1*	*Pax8*	TH response
Stomach	+++	+	-	-	No
Thyroid	+++	+++	+	+	Yes
Submandibular gland	++	+	-	-	No
Colon	+	+	-	-	No
Testis	+	++	-	-	No
Lung	+	+	+	-	Inverse
Liver	-	+	-	-	No
Pancreas	-	+	-	-	No

In the columns of *NIS *and *TSHR*, relative transcription levels obtained from end-point RTPCR are displayed as '+++', '++', '+' in descending order of expression, and negative expression is displayed as '-'. In the columns of *TTF1 *and *Pax8*, presence or absence of transcription are displayed as '+' and '-', respectively. Existence of thyroid-like responsiveness of *NIS *mRNA expression to thyroid hormone (up-regulated against methimazole and down-regulated against _L_-T_4_) is displayed as 'Yes', no-regulatory effect is displayed as 'No', and responsiveness in an inverse direction to thyroid (down-regulated against methimazole and up-regulated against _L_-T_4_) is displayed as 'Inverse' in the column of TH response. 'TH' stands for thyroid hormone.

## Conclusions

In this study, we carried out quantitative analysis of *NIS *mRNA in extrathyroidal tissues in different thyroid statuses for the first time to demonstrate that thyroid is the only tissue in which *NIS *mRNA level is inversely correlated with thyroid statuses, while *NIS *gene is widely expressed in extrathyroidal tissues. Absence of hypothyroidism-derived up-regulation and hyperthyroidism-derived down-regulation of *NIS *mRNA in extrathyroidal tissues seems to serve the purpose to concentrate iodide resource in thyroid tissue. Extrathyroidal *NIS *mRNA does not seem to be involved in iodide homeostasis.

## Competing interests

The authors declare that they have no competing interests.

## Authors' contributions

MHOR carried out end-point RTPCR, qPCR and RNA transfer to membrane for Northern blot. MA supervised all experimental procedures, drafted the manuscript, and drew figures and a table. XS carried out hybridization part of Northern blot hybridization. YH, JS, and YM made substantial contributions to conception of experiments and helped to draft the manuscript. All authors read and approved the final manuscript.
